# Comparative Study of Direct Resin Composites with Different Formulations

**DOI:** 10.3390/dj14080525

**Published:** 2026-08-17

**Authors:** Sufyan Garoushi, Enas Mangoush, Aous A. Abdulmajeed, Jasmina Bijelic-Donova, Pekka K. Vallittu, Lippo Lassila

**Affiliations:** 1 Department of Biomaterials Science, Turku Clinical Biomaterial Center—TCBC, Institute of Dentistry, University of Turku, 20520 Turku, Finland; enas.mangoush@utu.fi (E.M.); pekval@utu.fi (P.K.V.); liplas@utu.fi (L.L.); 2 Department of General Practice and Prosthodontics, School of Dentistry, Virginia Commonwealth University, Richmond, VA 23284, USA; aabdulmajeed@vcu.edu; 3 Department of Prosthetic Dentistry and Stomatognathic Physiology, Institute of Dentistry, University of Turku, 20520 Turku, Finland; jabije@utu.fi; 4 Wellbeing Services County of South-West Finland, 20521 Turku, Finland

**Keywords:** resin composite, filler loading, flexural properties, fracture toughness, surface properties

## Abstract

**Objectives**: The aim was to evaluate the mechanical, physical, and surface properties, as well as the microstructure, of three direct resin composites with different formulations (CLEARFIL AP-X, MAJESTY ES Flow, and MAJESTY ES-2) from the same manufacturer. **Methods**: Mechanical properties, including flexural strength, flexural modulus, and fracture toughness, were determined for each material (*n* = 8/material) according to ISO standards. Fourier transform infrared (FTIR) spectroscopy was used to determine the degree of conversion (DC%). Two-body wear was evaluated using a ball-on-flat configuration in a chewing simulator for 15,000 cycles, and wear depth was measured using a non-contact 3D optical profilometer. Polymerization shrinkage stress was measured using a tensilometer. Surface roughness and gloss were evaluated on disk-shaped specimens (*n* = 5/material) after polishing with 2400- and 4000-grit abrasive papers. Microstructural characterization was performed using scanning electron microscopy. Data were analyzed using ANOVA followed by Tukey’s HSD test (α = 0.05). **Results**: CLEARFIL AP-X exhibited the highest flexural strength (186.95 MPa), flexural modulus (19.25 GPa), and fracture toughness (1.50 MPa m^1/2^) among the tested materials (*p* < 0.05). However, it also showed the highest surface roughness and the lowest gloss values. MAJESTY ES Flow demonstrated the lowest wear depth and the highest gloss and polymerization shrinkage stress, whereas MAJESTY ES-2 exhibited the greatest wear and the lowest DC%. **Conclusions**: The three resin composites exhibited distinct mechanical, physical, surface, and microstructural characteristics associated with differences in their formulations, including filler content, filler morphology, and resin matrix composition. No single material demonstrated superior performance across all evaluated properties, highlighting the importance of selecting resin composites according to the specific clinical requirements.

## 1. Introduction

Developments in resin composites have focused on improving their durability, strength, and wear resistance, making them suitable for both anterior and posterior restorations. The mechanical, physical, and surface properties of resin composites, including flexural strength, fracture toughness, degree of conversion, polymerization shrinkage stress, wear resistance, surface roughness, and gloss, can vary significantly depending on their composition, particularly the filler characteristics and resin matrix structure [1]. For instance, flexural strength is primarily influenced by the filler content and the properties of the filler material [2,3], whereas polymerization shrinkage is mainly governed by the chemical composition of the organic matrix [1]. One approach to enhancing the performance of resin composites is the incorporation of nano- and submicron filler particles, resulting in nano-hybrid or micro-hybrid resin composites [4]. These systems facilitate higher filler loading while maintaining comparatively low viscosity, thereby improving handling, mechanical properties, and polishability [5]. Another approach that has emerged as an alternative to conventional nano- and micro-hybrid composites is the incorporation of nanoparticle aggregates or nanoclusters, which provide improved aesthetic and mechanical performance [6,7,8]. This enhancement has been attributed to the porous structure of the nanoclusters, which promotes stronger interaction with the resin matrix and more efficient distribution of mechanical stresses [6,7,8].

While filler loading is an important determinant of the mechanical performance of resin composites, its effect is closely influenced by other compositional factors, including filler morphology, particle size and distribution, filler–matrix interfacial bonding, silane treatment, and the composition of the resin matrix [1,2,3]. Consequently, the mechanical and physical properties of resin composites result from the combined effects of these variables rather than filler loading alone. In general, increasing filler loading is associated with improvements in compressive strength, flexural strength, and hardness [9,10], whereas polymerization shrinkage tends to decrease [11]. However, an optimal filler volume fraction may be reached, beyond which further increases in filler loading do not necessarily result in additional improvements [2]. For example, in specific resin composite systems, previous studies have reported that flexural strength and elastic modulus increased up to a filler volume fraction of approximately 60%, whereas fracture toughness reached a plateau at approximately 57%, with hardness showing a similar trend. These threshold values may vary depending on the overall composition and formulation of the resin composite [3,12].

Although filler characteristics are major determinants of resin composite performance, they represent only one component of a multifactorial material system. The composition of the organic resin matrix also plays a pivotal role in determining the properties of resin composites. Bis-GMA (bisphenol A-glycidyl methacrylate) and UDMA (urethane dimethacrylate) are widely used because of their high strength and rigidity [1]. However, their relatively high viscosity may limit handling characteristics. Therefore, reactive diluents such as TEGDMA (triethylene glycol dimethacrylate) are incorporated to reduce viscosity and improve monomer conversion [1]. Although the lower molecular weight and favorable stereochemistry of TEGDMA enhance polymerization efficiency, they also contribute to increased polymerization shrinkage and may adversely affect certain mechanical properties [13]. Consequently, the ratio of monomers and co-monomers, together with their viscosity and polymerization kinetics, has a substantial influence on the final characteristics of the composite [14]. A comprehensive understanding of how composite composition affects mechanical performance, polymerization efficiency, wear resistance, and surface characteristics is therefore essential for selecting the most appropriate restorative material for specific clinical applications.

Although numerous studies [2,4,15] have demonstrated that filler loading is an important determinant of the mechanical and physical properties of resin composites, most have investigated experimental formulations in which filler content was the only variable. Several previous studies comparing microhybrid, nanohybrid, and flowable resin composites have shown that differences in filler loading, filler morphology, and resin matrix composition can influence mechanical performance, polymerization behavior, wear resistance, and surface characteristics [4,15]. However, these investigations have generally focused on a limited number of properties or specific material formulations. Consequently, direct comparisons among contemporary commercially available resin composites with substantially different formulations remain limited. This is clinically relevant because clinicians are presented with a wide range of commercially available resin composites that are often recommended for similar restorative indications despite considerable differences in their composition. Although these materials may be indicated for the same clinical application, variations in filler loading, filler morphology, particle size, resin matrix composition, and viscosity can significantly influence their mechanical, physical, and aesthetic properties. Therefore, evaluating commercially available resin composites under identical experimental conditions provides clinically meaningful information for material selection and contributes to a better understanding of the relationship between composite composition and material performance. Such information may assist clinicians in selecting the most appropriate restorative material for different clinical situations by balancing requirements for strength, wear resistance, polymerization behavior, and surface quality.

Based on this rationale, the present in vitro study evaluated three commercially available resin composites from the CLEARFIL series (Kuraray, Tokyo, Japan): CLEARFIL AP-X, CLEARFIL MAJESTY ES Flow, and CLEARFIL MAJESTY ES-2. Although all three materials are intended for direct restorative applications, they differ in viscosity, filler loading, filler morphology, and other compositional and structural characteristics. According to the manufacturer’s specifications, CLEARFIL AP-X is a microhybrid packable composite with a high filler loading (86 wt.%), CLEARFIL MAJESTY ES Flow is a flowable composite containing 75 wt.% fillers, and CLEARFIL MAJESTY ES-2 is a nanohybrid composite with a filler loading of 78 wt.%. In addition, the resin matrix formulations of the tested materials are not identical, and differences in monomer composition may also contribute to variations in their polymerization behavior and mechanical, physical, and surface properties. To our knowledge, no previous study has comprehensively compared the flexural properties, fracture toughness, degree of conversion, polymerization shrinkage stress, wear resistance, surface roughness, and gloss of these three commercially available CLEARFIL resin composites under identical experimental conditions. Such a comprehensive evaluation provides new insight into how differences in filler loading, filler morphology, particle size, resin matrix formulation, and viscosity collectively influence material performance and may assist clinicians in selecting restorative materials for different clinical situations.

Therefore, the aim of this study was to compare the flexural properties, fracture toughness, degree of conversion, polymerization shrinkage stress, wear resistance, surface roughness, and gloss of commercially available resin composites with different formulations and to evaluate how differences in their overall composition influence material performance. The null hypothesis was that the type of resin composite would have no significant effect on these properties.

## 2. Materials and Methods

Resin composites used in this study are listed in Table 1. The examiner performing all experimental measurements and analyses was aware of the material group allocation (i.e., was not blinded). The sample size for each experiment was determined based on previously published in vitro studies with similar experimental designs and in accordance with our laboratory’s established testing protocol [1,2,16].

### 2.1. Flexural Strength and Modulus

For the three-point bending test, 48 specimens of specific dimension (2 × 2 × 25 mm^3^) were prepared from the three tested materials (*n* = 16/material). By using a half-split stainless-steel mold and transparent Mylar sheets, bar-shaped specimens were prepared. Light curing of resin composite was performed by a hand light-curing unit (Elipar DeepCure-L, 3M, Maplewood, MN, USA) for a duration of 20 s in four different parts through metal molds on both sides. The curing device’s light tip was 1 mm away from the resin composite. The light intensity was 1600 mW/cm^2^, with a wavelength ranging from 400 to 480 nm (Marc Resin Calibrator, BlueLight Analytics Inc., Halifax, NS, Canada). The light output of the curing unit was measured and verified at the beginning of the study. Before testing, the specimens of each material were divided into two subgroups (*n* = 8/subgroup) and each subgroup was tested under different conditions. This includes testing after 48 h of dry storage in an incubator at 37 °C, and testing following hydrothermal accelerated aging in boiling water for 16 h. This accelerated aging protocol has been used in dental materials research as a rapid alternative to long-term water storage [17,18]. The three-point bending test was conducted following the ISO 4049 standard [19]. The test setup included a 20 mm span, a loading speed of 1 mm per minute, a 2 mm diameter indenter, and a 2500 N load cell. All specimens were tested using a material-testing machine (model LRX, Lloyd Instruments Ltd., Fareham, UK). The load-deflection curves were recorded using PC software (Nexygen 4.0, Lloyd Instruments Ltd., Fareham, UK).

Flexural strength (ơf) as well as flexural modulus (Ef) were measured according to equations:

ơf = 3FmI/(2bh^2^)

Ef = SI^3^/(4bh^3^)

where Fm is the load applied (N) at the maximum point of the load-extension curve, I is the length of span (20 mm), b is the test specimens’ width and h represents the test specimens’ thickness. S is the stiffness (N/m) S = F/d where d represents the deflection that corresponds to load F at a particular point along the straight-line portion of the trace.

### 2.2. Fracture Toughness

In accordance with the ISO 20795-2 standard method (ASTM 2005) [20], single-edge-notched-beam specimens (2.5 × 5 × 25 mm^3^) were prepared to determine fracture toughness. A half-split stainless steel mold was used, allowing removal of the specimens without applying force. A centrally positioned slot was incorporated into the mold and extended to the mid-height of the specimen, ensuring a central notch position and optimizing the crack length (x) to approximately half the specimen height. The composite resin was packed into the mold in a single increment placed over a Mylar-strip-covered glass slide. A sharp and centrally positioned notch was created prior to polymerization by inserting a straight-edged steel blade into the prefabricated slot. The notch geometry and tip sharpness were verified before testing according to the same methodology used in previous studies [16]. Polymerization was performed according to the previously described light-curing protocol. Before polymerization, the upper surface of the mold was covered with a Mylar strip and a glass slide on both sides of the blade. After removal from the mold, the opposite side of each specimen was additionally polymerized. Before testing, the specimens from each group (*n* = 8) were stored dry in an incubator (37 °C) for 48 h. The specimens were tested in a 3-point bending mode with a crosshead speed of 1.0 mm/minute on a universal material testing machine. The fracture toughness was measured through the following formula:

$$K.\;max=[(P\cdot L)/(B\;W^{3⁄2})]\;f(x)$$

where f is a geometrical function dependent on x:

$$f\left(x\right)=3x^{1/2}[1.99-x\left(1-x\right)(2.15-3.93x+2.7x^{2})]\left[2\left(1+2x\right){\left(1-x\right)}^{3/2}\right]$$

Here, L is the length of span (2 cm), B is the thickness of specimen in centimeters (cm), P is the maximum load in kilonewtons (kN), W is the width of specimen (depth) in cm, x is a geometrical function dependent on a/W and a is the length of crack in cm.

### 2.3. Degree of Monomer Conversion DC%

The DC% was assessed using Fourier-transform infrared spectroscopy (FTIR) with an attenuated total reflectance (ATR) accessory (Spectrum One, Perkin-Elmer, Beaconsfield Bucks, UK). Resin composites were placed in a mold measuring 2 mm in thickness and 4 mm in diameter for analysis. Initially, the spectrum of the unpolymerized specimen was recorded. Subsequently, specimens were polymerized according to the previously described light-curing protocol through a glass slide. FTIR measurements were performed on the same specimen at 3, 6, 9, 12, and 15 min after photoactivation, allowing the changes in degree of conversion over time to be monitored. Each specimen (*n* = 5) underwent five repetitions of this procedure. The DC% was determined by evaluating the aliphatic C=C peak at 1636 cm^−1^, which was then normalized against the carbonyl C=O peak at 1720 cm^−1^, considering the absence of aromatic peak C=C in certain resin composites. The normalized absorbance values for the uncured ($\frac{C=C_{uncured}}{C=O_{uncured}}$) and cured ($\frac{C=C_{cured}}{C=O_{cured}}$) specimens were employed in the calculation of the degree of conversion using the following formula:

$$DC\%=100\times (1-\frac{C=C_{cured}∕C=O_{cured}}{C=C_{uncured}∕C=O_{uncured}})$$

### 2.4. Two-Body Wear

The two-body wear test was applied according to previous studies [14,18] to evaluate the wear resistance of each resin composite. Two polished specimens (4000-grit papers) (20 mm length × 10 mm width × 3 mm depth) of each material were prepared in acrylic resin blocks. All specimens were immersed in distilled water at 37 °C for 48 h prior to testing. The wear assessments were conducted using a chewing simulator (CS-4.2, SD Mechatronik, Feldkirchen-Westerham, Germany), consisting of two distinct chambers designed to replicate vertical and horizontal masticatory movements sequentially while operating within an aqueous environment. Each chamber consisted of a lower sample holder for specimen insertion and an upper loading tip serving as the counteracting element. The specimens were centrally positioned within the chamber to ensure a standardized testing configuration and minimize potential positional effects during the wear analysis. An upper antagonist, in the form of a steatite ball with a 6 mm diameter, was employed. For each specimen, a total of 15,000 simulated chewing cycles were applied at a frequency of 1.5 Hz, using a vertical load of 2 kg, corresponding to a chewing force of 20 N. After the simulations, wear patterns were assessed using a 3D optical profilometer (ContourGT-I, Bruker Nano, Inc., Tucson, AZ, USA). The average wear depth (in µm) was calculated based on the deepest points identified from six wear profiles per group, providing a comprehensive measure of wear resistance.

### 2.5. Shrinkage-Stress Measurement

Glass fiber-reinforced composite (FRC) rods (length: 4 cm; diameter: 4 mm) were ground flat at their ends using 500-grit silicon carbide paper. The FRC rods were then tightly attached to the universal testing machine, and the resin composite was placed between the two opposing rod surfaces (*n* = 5/material). The specimen height was standardized to 1.5 mm. The tip of the light guide of the light-curing unit (Elipar) was positioned in close contact with both sides of the composite specimen during polymerization. No adhesive or primer was applied to the FRC rod surfaces prior to resin composite placement. At room temperature (22 °C), contraction forces were continuously monitored for 3 min. Shrinkage stress was calculated by dividing the measured contraction force by the cross-sectional area of the FRC rod. At the end of the shrinkage stress–time curve, the maximum shrinkage stress (SS) value was determined from the plateau region.

### 2.6. Surface Gloss

Disk-shaped specimens (15 mm diameter and 2 mm thickness) were fabricated from each material using a metal mold (*n* = 5/material). One side of each specimen, corresponding to the surface facing the mold, was polished flat using silicon carbide papers (2400-grit and 4000-grit) under water cooling at 300 rpm with an automatic grinding machine (Rotopol-1; Struers, Copenhagen, Denmark). This surface was designated as the polished side. The other side of the specimens facing the glass slide and mylar strip remained unpolished and was named the unpolished side. After polishing, specimens were rinsed with water and stored dry at room temperature before testing. Surface gloss was measured at a 60° incidence angle using a calibrated infrared Zehntner Glossmeter (GmbH Testing Instruments, Solingen, Germany) with a square measurement area of 6 mm × 40 mm. The glossmeter was calibrated immediately before the measurements according to the manufacturer’s instructions, and all measurements were performed by the same examiner. The average of four measurements was recorded for each specimen surface.

### 2.7. Surface Roughness

The surface roughness of each disk-shaped specimen was assessed before and after polishing using a 3D non-contact optical profilometer (Bruker Nano GmbH Berlin, Germany). A 5× objective lens and a 0.5 multiplier were employed, with a back scan and length parameter of 20 μm and 60 μm, respectively, in VSI/VXI mode to capture a 3D representation of the specimen surfaces (*n* = 5/material). The Vision 64 software (version 1, Bruker Nano GmbH, Berlin, Germany) was utilized to generate roughness and surface area parameters, with Ra (roughness average) being the primary parameter of interest, measured from a mean line within the sampling length.

### 2.8. Microstructure Analysis

The microstructure of the investigated resin composites was evaluated using scanning electron microscopy (SEM) in backscattered electron (BSE) mode and energy-dispersive spectroscopy (EDS) (LEO, Oberkochen, Germany). BSE imaging was used to assess the filler morphology and distribution, while EDS was used to determine the elemental composition of the filler particles. Polished disk-shaped specimens (*n* = 2/material) were stored dry in a desiccator for 48 h and subsequently sputter-coated with gold using a vacuum sputter coater (BAL-TEC SCD 050, Balzers, Liechtenstein). SEM images were acquired in BSE mode at magnifications of 500×, 1500×, 2500×, and 5000× using an accelerating voltage of 20 kV and a working distance of 13.1 mm. EDS analysis was carried out under the same operating conditions.

### 2.9. Statistical Analysis

Analysis of variance (ANOVA) with a significance level of 0.05 was employed to statistically analyze the data using SPSS version 29 (IBM Corp., Armonk, NY, USA). The normality of the data distribution was assessed using the Shapiro–Wilk test, and homogeneity of variances was evaluated using Levene’s test. Two-way ANOVA was used to evaluate the effects of material type, aging, and their interaction on flexural strength and flexural modulus. One-way ANOVA was used to analyze fracture toughness, degree of conversion, wear depth, gloss, surface roughness, and polymerization shrinkage stress. Tukey’s HSD post hoc test was applied for pairwise comparisons when significant differences were detected.

## 3. Results

**Figure 1:**
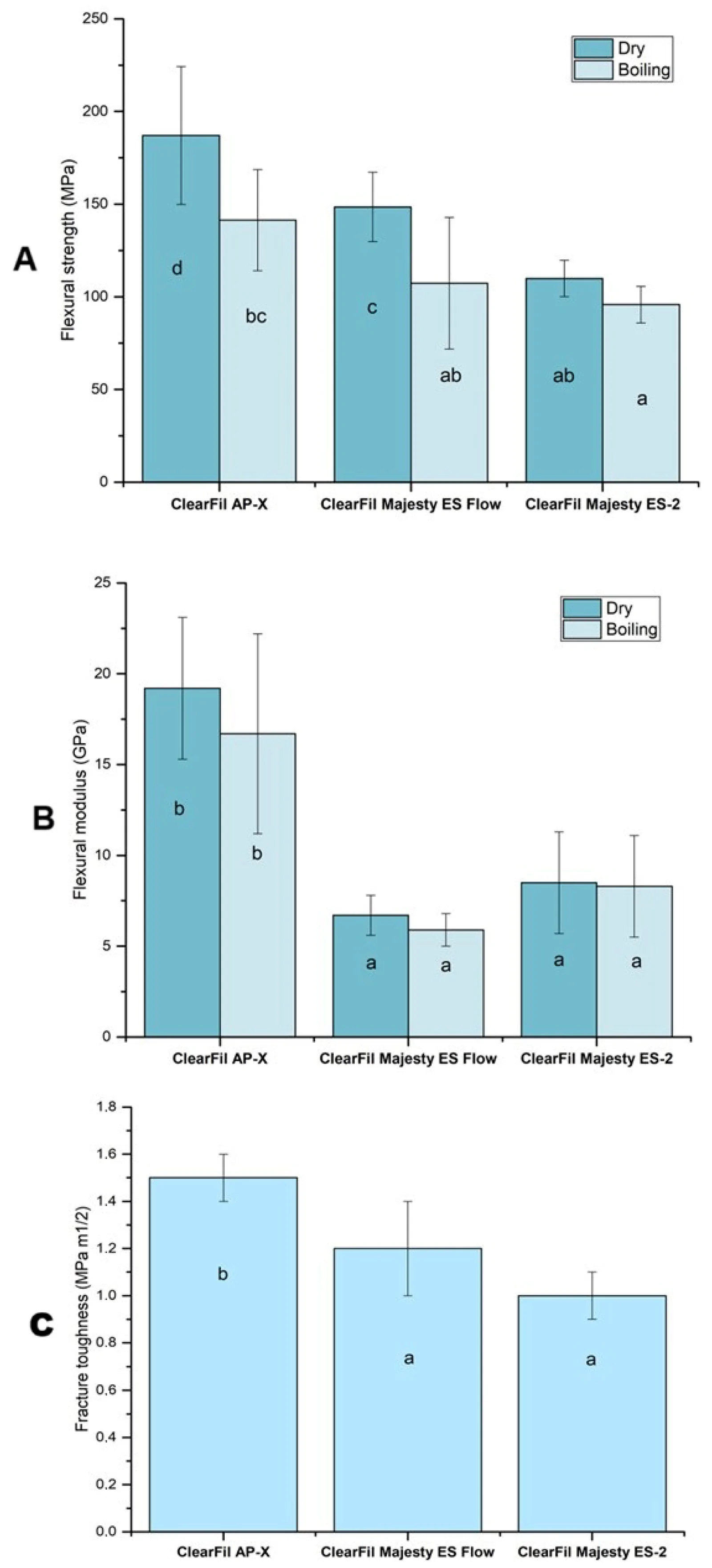
The mean values of (**A**) flexural strength (MPa), (**B**) flexural modulus (GPa) and (**C**) fracture toughness (MPa·m^1/2^) of the investigated resin composites. Different letters indicate statistically significant differences (*p* < 0.05).

Flexural strength and modulus mean values of the tested materials before and after hydrothermal accelerated aging are presented in Figure 1A,B. A two-way ANOVA revealed that both material type (*p* = 0.003) and aging (*p* < 0.001) significantly affected flexural strength, whereas only material type significantly affected flexural modulus (*p* < 0.001), with no significant effect of aging (*p* = 0.220). The interaction between material type and aging was not statistically significant for either flexural strength (*p* = 0.183) or flexural modulus (*p* = 0.600), indicating that the effect of aging on these flexural properties was comparable among the tested materials. Hydrothermal accelerated aging decreased the flexural properties of all three tested resin composites. AP-X specimens tested after dry storage showed the highest (*p* < 0.05) flexural strength (186.95 ± 37 MPa) and modules (19.25 ± 4 GPa) values. One-way ANOVA and Tukey’s HSD test revealed that AP-X group showed the highest fracture toughness result (1.5 ± 0.1 MPa m^1/2^) and the difference compared to the other two materials was statistically significant (*p* < 0.05) (Figure 1C), while ES-2 group showed the lowest fracture toughness values (1.01 ± 0.1 MPa m^1/2^); however, this difference was not significantly different (*p* > 0.05) compared to the ES-Flow (1.22 ± 0.2 MPa m^1/2^).

**Figure 2:**
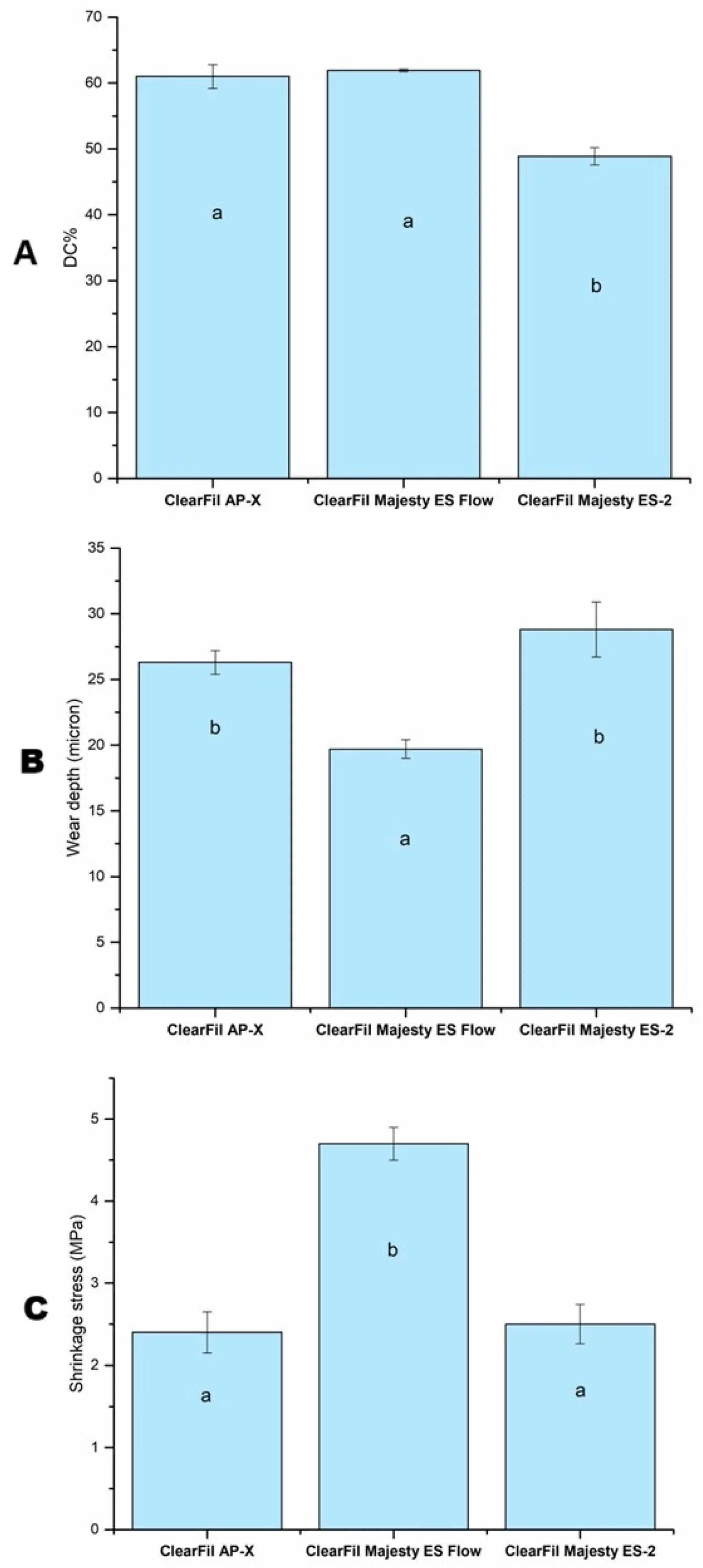
The mean values of (**A**) degree of conversion (DC%), (**B**) wear depth (μm) and (**C**) shrinkage stress (MPa) of the investigated resin composites. Different letters indicate statistically significant differences (*p* < 0.05) among the tested materials.

The DC% values are presented in Figure 2A. ES-Flow (61.9 ± 1.3%) and AP-X (61.0 ± 0.2%) exhibited comparable DC% values, with no statistically significant difference between them (*p* > 0.05). In contrast, ES-2 showed the lowest DC% (48.9 ± 1.8%), which was significantly lower than those of ES-Flow and AP-X (*p* < 0.05). Wear depth results following 15,000 chewing simulation cycles of the two-body wear test are shown in Figure 2B. ES-Flow specimens exhibited the lowest mean wear depth (19.7 ± 0.7 μm), which was significantly lower (*p* < 0.05) than those of AP-X and ES-2 (26.3 ± 0.9 and 28.8 ± 2.1 μm, respectively). No statistically significant difference in wear depth was observed between AP-X and ES-2 (*p* > 0.05). Figure 2C presents the shrinkage stress values for each material. ES-Flow exhibited the highest shrinkage stress (4.7 ± 0.2 MPa), which was significantly greater than that of both AP-X and ES-2 (*p* < 0.05). In contrast, AP-X and ES-2 exhibited shrinkage stress values of 2.4 ± 0.2 MPa and 2.5 ± 0.2 MPa, respectively, with no statistically significant difference between them (*p* > 0.05).

**Figure 3:**
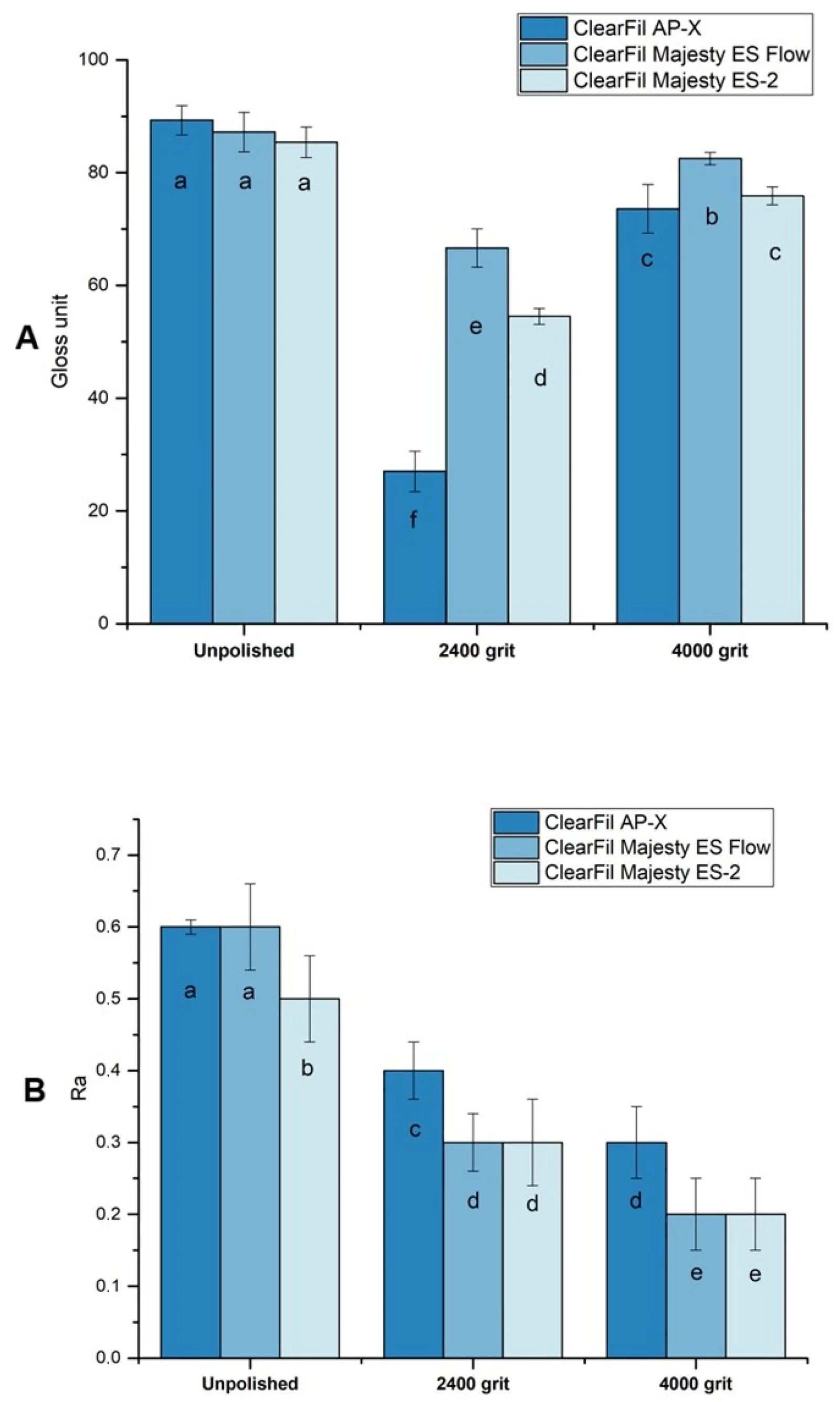
The mean values of (**A**) surface gloss and (**B**) surface roughness (μm) of the investigated resin composites before and after polishing with different silicon carbide paper grit sizes (unpolished, 2400, and 4000). Different letters indicate statistically significant differences (*p* < 0.05) among the tested materials.

Figure 3A presents the gloss values of the tested materials following baseline (unpolished), 2400-grit, and 4000-grit polishing. Two-way ANOVA revealed that both material type and polishing condition significantly affected gloss (*p* < 0.05). In addition, a significant interaction between material and polishing condition was observed (*p* < 0.05), indicating that the effect of polishing varied among the tested materials. Across all materials, the unpolished specimens exhibited the highest gloss values (89.3 ± 2.6, 87.2 ± 3.5, and 85.4 ± 2.7 GU for AP-X, ES-Flow, and ES-2, respectively). Polishing with 2400-grit paper resulted in a marked decrease in gloss for all materials, with AP-X showing the lowest gloss value (27 ± 3.6 GU) compared with ES-Flow (66.6 ± 3.4 GU) and ES-2 (54.5 ± 1.4 GU). Subsequent polishing with 4000-grit paper increased the gloss values relative to the 2400-grit condition (73.6 ± 4.3, 82.5 ± 1.1, and 75.9 ± 1.6 GU for AP-X, ES-Flow, and ES-2, respectively). Unpolished specimens exhibited the highest surface roughness values (Figure 3B). Polishing with 2400-grit sandpaper reduced the surface roughness of all tested materials relative to the unpolished specimens. Further polishing with 4000-grit sandpaper resulted in a marked reduction in roughness values. AP-X specimens showed the highest roughness post-polishing with 4000-grit (0.3 ± 0.05 µm) compared to their unpolished value of 0.6 ± 0.01 µm. In contrast, ES-Flow and ES-2 displayed similar roughness values of 0.2 µm, down from 0.6 ± 0.06 µm and 0.5 ± 0.06 µm, respectively, prior to polishing.

**Figure 4:**
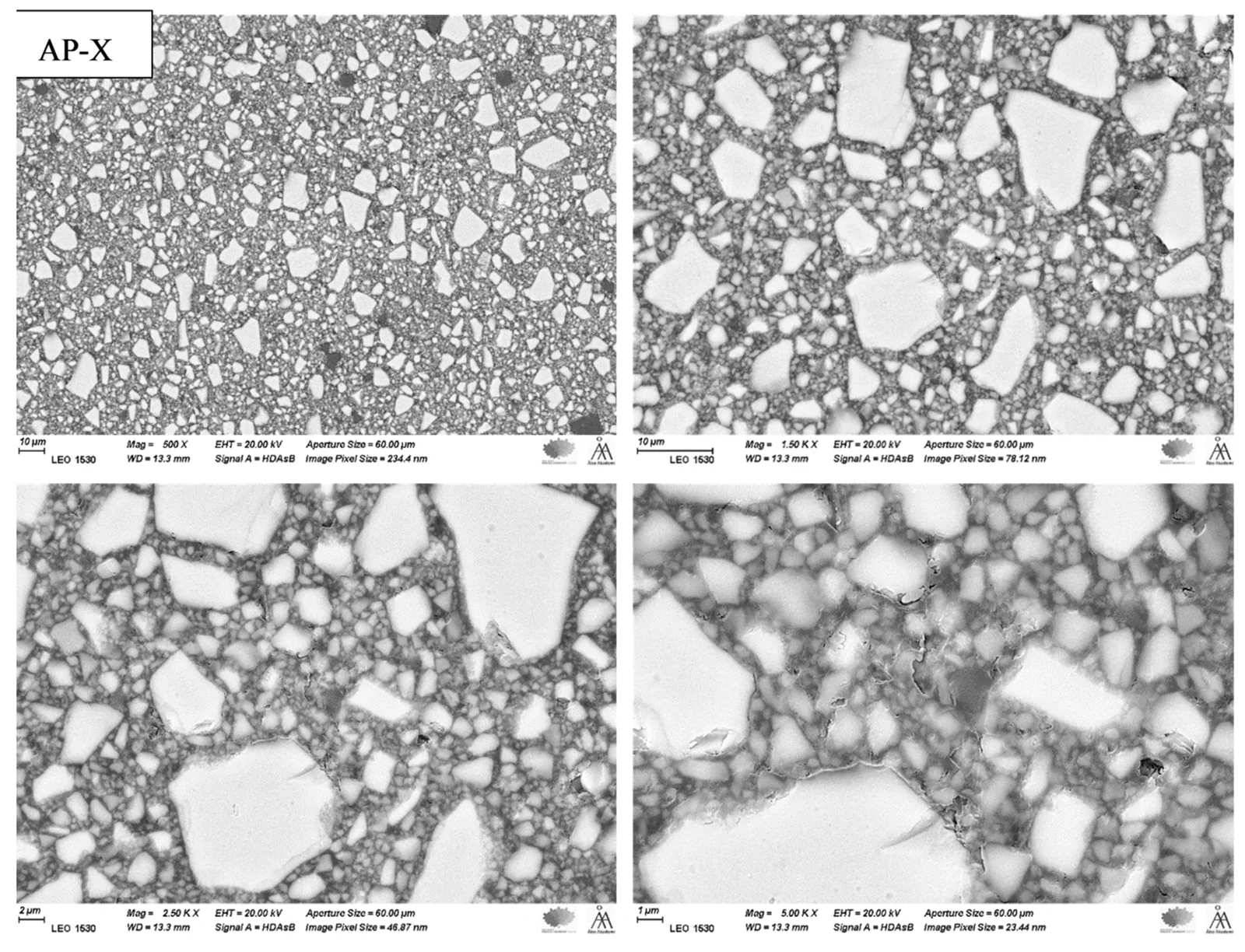
SEM images of AP-X resin composite (under 500, 1500, 2500, and 5000× magnification).

**Figure 5:**
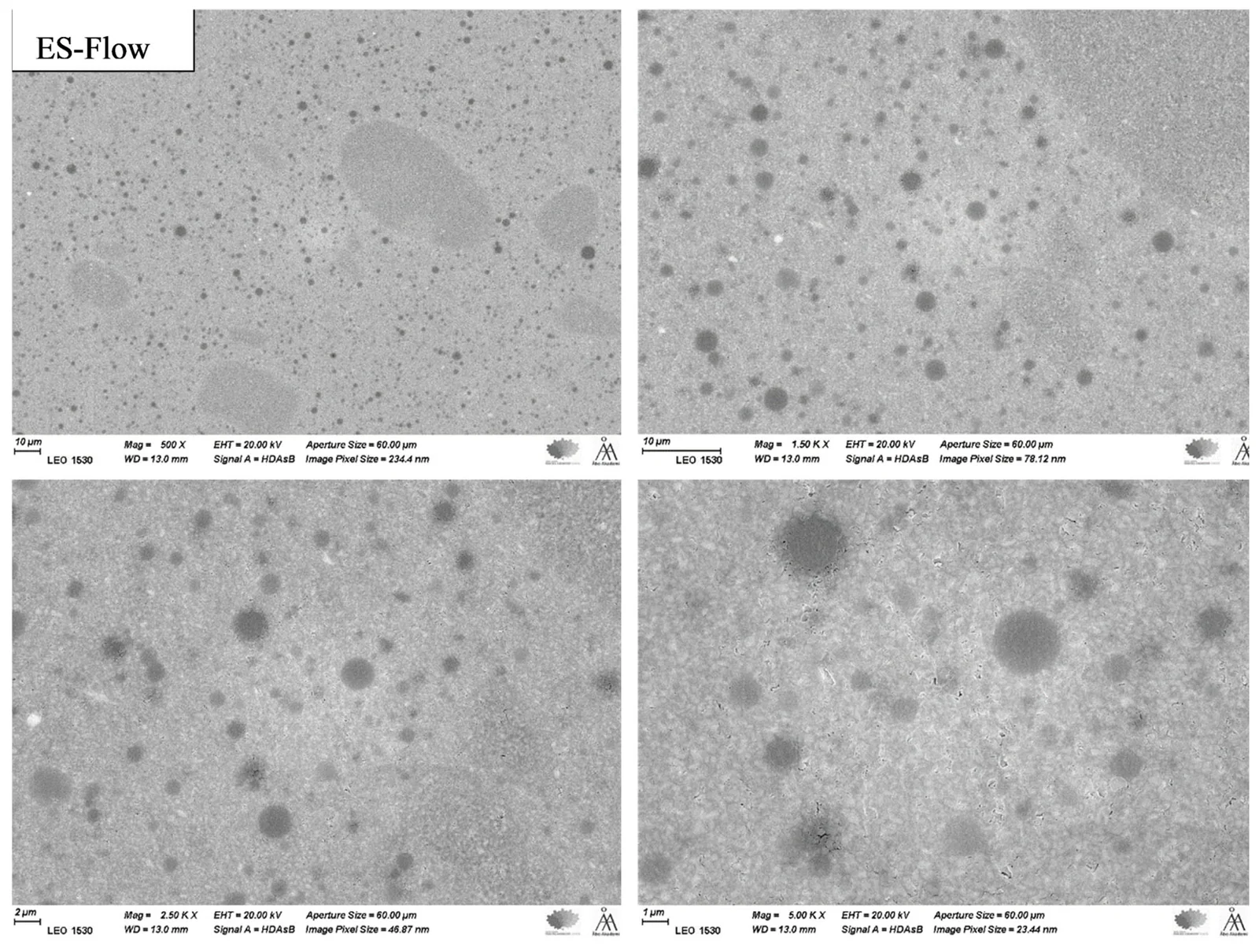
SEM images of ES-Flow resin composite (under 500, 1500, 2500, and 5000× magnification).

**Figure 6:**
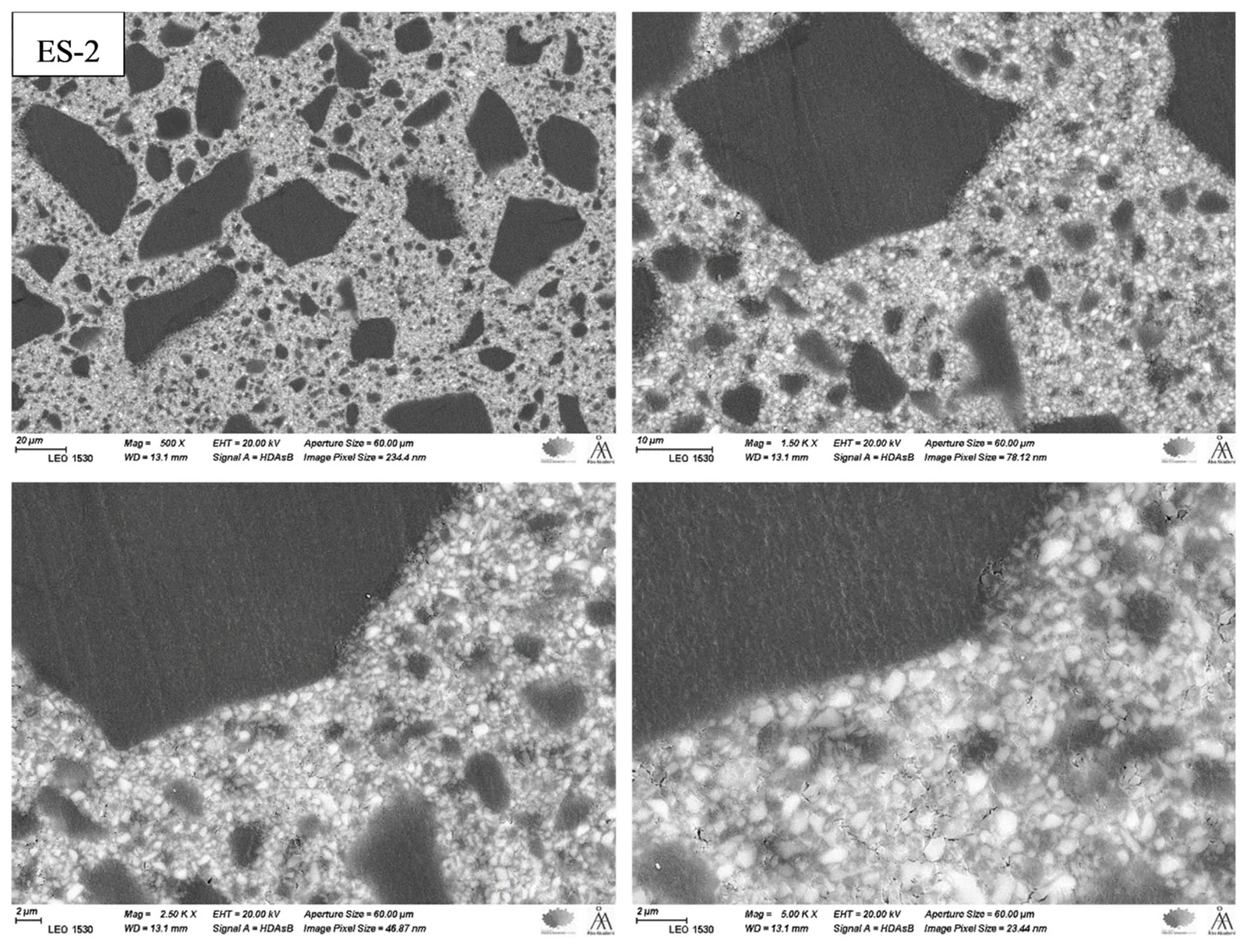
SEM images of ES-2 resin composite (under 500, 1500, 2500, and 5000× magnification).

The major elemental composition of the tested materials, as determined by EDS, is presented in Table 2. SEM analysis revealed distinct microstructures for each tested resin composite, characterized by variations in filler shape and size within the composite matrix (Figure 4, Figure 5 and Figure 6). AP-X exhibited filler sizes ranging from sub-micron levels to over 10 µm. In contrast, ES-Flow displayed a combination of submicron fillers and clustered fillers, some exceeding tens of microns in size. Nano-hybrid ES-2 demonstrated a broader range of filler sizes, with some particles size exceeding 20 µm. These larger structures represent pre-polymerized filler clusters.

## 4. Discussion

The overall clinical success of resin composites is multifactorial in nature. One of the primary causes of failure of direct resin composite restorations is material fracture [21,22], making the assessment of mechanical properties essential. Flexural testing is commonly utilized for this purpose, along with the evaluation of fracture toughness to understand a material’s resistance to crack propagation [23]. Additionally, wear resistance is a critical mechanical property that should be assessed, especially for materials intended for use on occlusal surfaces, where mechanical forces are in their highest level [24]. Physical properties are equally important to consider. For instance, shrinkage stress during polymerization is a key factor, as it directly impacts the marginal seal of the restoration and can influence long-term clinical outcomes [25]. Hence, these critical properties were evaluated in our study to provide a comprehensive understanding of the mechanical and physical performance of the tested resin composites.

Our results clearly demonstrated that the tested resin composites exhibited distinct mechanical, physical, and surface properties, reflecting differences in their overall formulations, including filler content, filler morphology, and resin matrix composition.

AP-X exhibited flexural strength (186.95 MPa) and flexural modulus (19.25 GPa) under dry conditions, revealing superior flexural properties compared to ES-Flow and ES-2. This may be attributed to the distinct filler load, composition, size, distribution, and organic matrix structure. Previous research [2,15] indicated a positive correlation between the percentages of filler content and the mechanical properties, specifically the flexural strength. These studies demonstrated a direct proportional relationship, whereby an increase in filler content corresponded to an increase in flexural strength. A possible explanation for this phenomenon could be provided through an examination of the quantity of inter-particle contacts that facilitate the transmission and diffusion of forces throughout the material. Rather than the stress being localized at a singular point, which renders it susceptible to failure, this stress is distributed and transmitted across multiple adjacent particles. AP-X contained the highest filler load (86 wt.%) compared to the other tested materials; therefore, this could be one of the possible explanations for the acquired results. Nonetheless, while this correlation aligns effectively with the findings from the flexural modulus analysis, wherein ES-2, which has a higher filler content (78 wt.%), exhibited elevated flexural modulus values compared to ES-Flow (75 wt.%), it does not sufficiently explain the observed comparative flexural strength results for these two materials. Our data indicates that ES-Flow demonstrates superior flexural strength values relative to ES-2. This variation highlights the potential influence of the size of the fillers and the organic matrix as critical factors contributing to the determination of flexural properties. For instance, as reported by the manufacturer, ES-Flow comprises submicron and clustered fillers with sizes ranging from 0.18 to 3.5 µm, whereas ES-2 is characterized as a nano-hybrid composite with fillers size ranging from 1 to 100 nm in addition to other bigger micrometric particles (Figure 4, Figure 5 and Figure 6). While the filler load between the two materials does not exhibit significant variation, the larger particle sizes in this case would result in resin composites with superior flexural strength. These findings align with prior research that presented nearly similar findings to the results obtained in this study [26,27]. The structure of the organic matrix also plays a crucial role in determining the mechanical properties. Various studies have examined differences between organic matrices containing different resin monomers [28,29,30,31].

According to our findings, hydrothermal accelerated aging significantly (*p* < 0.05) diminished the flexural strength of all tested resin composites, while its effect on the flexural modulus appears to be less prominent (*p* > 0.05). These results are in accordance with prior research, which indicated that flexural strength is influenced by the aging process, whereas modulus is either less impacted or remains totally unaffected [32,33]. Effects of the hydrothermal accelerated aging on the flexural strength could be attributed primarily to mechanisms related to water uptake that causes matrix expansion and internal stresses [18]. Hydrothermal accelerated aging is a method used to simulate how a material will perform over time under normal usage conditions. It involves exposing the material to moisture and thermal stresses that are more extreme than those typically experienced in the environment, but for a short period of time. While this laboratory aging process does not perfectly replicate real-life conditions, it helps provide an indication of the material’s long-term stability [18]. Exposure to boiling water promotes water penetration into the resin matrix, softening the material and inducing hydrolysis of the silane bonding agent at the matrix-filler interface. This results in hydrolytic degradation and weakens chemical bonds between the resin and filler particles, further reducing the flexural strength of the material [17,34].

The two-way ANOVA analysis confirmed that both the type of resin composite and aging conditions played a significant role in determining the flexural properties of the materials. Moreover, the effect of aging seems to be material-dependent, for instance, ES-2 showing more resistance to aging than the AP-X and ES-Flow groups (Figure 1). This observation aligns with earlier research, which has demonstrated that the response of various materials to hydrothermal accelerated aging can differ significantly depending on their structure [35,36].

In terms of fracture toughness, AP-X again showed superior performance with the highest (*p* < 0.05) values (1.5 MPa m^1/2^), which was significantly higher than ES-Flow and ES-2. One possible justification of the acquired result could be as mentioned previously because of the inter-particle distance of fillers, along with the concentration and size of the filler particles, and the enhanced bond at the filler-matrix interface, constitutes a critical parameter for optimizing the fracture toughness. Moreover, AP-X contains fillers that varies greatly in size (0.1–15 μm), the integration of fillers with heterogenous sizes within a polymer matrix impedes or necessitates greater energy for the initiation, growth, and propagation of cracks [3].

ES-2, which showed the lowest fracture toughness values, is the only resin composite included in the current study that contains pre-polymerized organic fillers. According to the findings of Nicoleta and Khaund et al., the bond between pre-polymerized fillers and the organic matrix is considered to be weaker than the bond established between the inorganic fillers and the matrix, which could result in the obtained values [3,37]. The influence of organic matrix structure is also significantly evident in the fracture toughness results. Sideridou et al. have shown in their research that a reduction in fracture toughness is anticipated in formulations with elevated filler content and a higher ratio of Bis-GMA, such as ES-2. This is attributable to the increased brittleness of these systems, which consequently results in diminished fracture toughness and a heightened susceptibility to catastrophic failure. In contrast, AP-X incorporates TEGDMA alongside Bis-GMA, serving as a diluent that enhances both the handling characteristics and mechanical properties of the resin composite [13].

The analysis of wear resistance revealed notable differences among the evaluated resin composites. Despite having the lowest filler content, ES-Flow exhibited the shallowest wear depth (19.7 μm) following 15,000 cycles of chewing simulation, outperforming both AP-X (26.3 μm) and ES-2 (28.8 μm). These findings differ from previous studies reporting a positive correlation between filler loading and wear resistance [38,39,40], suggesting that factors other than filler content may also contribute to wear behavior. Such factors may include filler morphology, filler composition, particle size, and resin matrix composition. However, since the present study was not designed to evaluate the individual contribution of these variables, their specific influence cannot be confirmed and should be interpreted with caution. Previous studies have suggested that the improved wear resistance associated with TEGDMA-containing resin matrices may be related to their higher degree of conversion and greater flexibility, which could enhance energy absorption during wear [41]. Likewise, Suzuki et al. proposed that spherical fillers may exhibit superior wear resistance compared with irregularly shaped fillers [42]. In the present study, SEM observations (Figure 4, Figure 5 and Figure 6) showed that ES-Flow contained predominantly spherical fillers, whereas AP-X and ES-2 exhibited more irregularly shaped fillers. Although these compositional and microstructural differences may have contributed to the superior wear resistance of ES-Flow, the present findings do not allow the individual effects of filler morphology or resin matrix composition to be distinguished. In contrast to the present findings, two recent studies by Osiewicz et al. reported no significant differences in wear between CLEARFIL AP-X and CLEARFIL MAJESTY ES-2, while CLEARFIL MAJESTY ES Flow exhibited greater wear depth than the packable CLEARFIL AP-X [43,44]. This discrepancy may be attributed to differences in the wear-testing methodology, experimental conditions, and wear evaluation protocols employed in the two studies.

The kinetics of shrinkage stress exhibit complicated dynamics influenced by numerous contributing variables. Like previous properties, these variables include the shape and size of filler particles, the structural characteristics of the organic matrix, and the pivotal roles played by the degree of conversion and shrinkage strain. Modifications to any of these parameters may affect the resultant shrinkage stress values [45]. Moreover, other factors in addition to the materials structure could also play a role to determine the shrinkage stress, for instance, the reconstruction and curing methods, and the C-factor [46]. ES-Flow demonstrated the highest shrinkage stress (4.7 MPa), almost twice that of AP-X and ES-2. The results can be easily justified by its reduced volume of filler content. Julian et al. have demonstrated that a reduction in filler particle size results in an increased surface area of the dispersed phase, subsequently enhancing the constraints imposed on the matrix phase; thus, shrinkage stress is inversely proportional to filler particle size [45]. According to our findings, despite ES-2 exhibiting the smallest filler size, its shrinkage stress is lower than that of ES-Flow, prompting consideration of additional factors that may offset, either completely or partially, the effects of reduced filler size. Notably, the higher volume content of fillers plays a significant role, and the inclusion of prepolymerized fillers has been demonstrated to effectively reduce shrinkage stress [38,46,47]. Furthermore, the type of monomer used is a critical determinant; TEGDMA, the predominant monomer in ES-Flow, possesses a low molecular weight and viscosity, which can lead to elevated shrinkage stresses due to its heightened reactivity, resulting in increased conversion and consequently higher shrinkage [46,48,49]. Conversely, the advantages of using Bis-GMA over other small-sized monomers include a reduction in shrinkage stress, a finding confirmed by our results [38].

The gloss and surface roughness results generally followed the expected inverse relationship, with increasing polishing grit size resulting in higher gloss and lower surface roughness. However, an exception was observed for the unpolished specimens, which exhibited relatively high gloss despite their greater surface roughness. This behavior can be attributed to the resin-rich surface layer formed directly against the mold, which remained relatively homogeneous and reflected light efficiently despite the higher Ra values. These findings suggest that gloss is influenced not only by surface roughness but also by surface topography and the optical characteristics of the outer resin-rich layer. Overall, the principal determinant of gloss and surface roughness was the grit size of the silicon carbide polishing papers; increasing grit size produced smoother surfaces with higher gloss. These results are consistent with previous studies reporting an inverse relationship between gloss and surface roughness following polishing [50,51,52]. Among the tested groups, the submicron-filled ES-Flow and nanohybrid ES-2 demonstrated higher gloss values than the microhybrid AP-X following polishing with 2400- and 4000-grit silicon carbide papers. These findings contrast with those reported by Kaizer et al., who concluded that there is insufficient evidence to support the assertion that nano- and submicron-filled resin composites yield superior gloss compared with microhybrid composites [53]. On the other hand, Islam et al. reported that CLEARFIL AP-X exhibited gloss values comparable to those of other conventional nanohybrid resin composites [54], further highlighting the inconsistent findings in the literature. These discrepancies may be attributed to differences in material formulations, polishing protocols, and gloss measurement methodologies. Surface gloss is also influenced by the type, loading, and distribution of fillers, as well as their optical properties, which may vary among the materials investigated in different studies [55,56].

The application of 4000-grit paper produced optimal results across all examined materials when assessing both gloss and surface roughness. This grit size facilitated surface roughness measurements within the range of 0.2 to 0.3 µm, as evidenced by clinical trials and in vitro studies, which demonstrate that these levels are below the thresholds associated with plaque accumulation and periodontal complications [57,58]. Moreover, it results in gloss values exceeding 70 GU, which is regarded as an excellent finish, positively surpassing the clinically acceptable threshold values for gloss [55,59].

The specimens’ analysis via SEM/EDS provided further insights into the differences in the materials’ structure. EDS analysis revealed comparable concentrations of Al and Si across all tested materials. However, higher concentrations of Ba were detected in the AP-X specimens compared to the other two materials, which is attributed to the greater filler concentration in this material. Moreover, elevated levels of C and N were observed in the ES-2 specimens, which may arise not only from its pre-polymerized organic filler content but also from factors such as differences in the organic matrix component, surface contamination and specimens’ heterogeneity.

It is essential to consider the limitations of this study and future research directions to further elucidate the properties and differences among the investigated materials. For instance, water sorption and solubility, color stability, and volumetric shrinkage are important properties that warrant further investigation. In addition, all tested materials were produced by a single manufacturer, which may limit the generalizability of the findings to resin composites from other manufacturers. Another limitation is that the filler contents of the investigated materials were based on the manufacturer’s specifications and were not independently verified experimentally. Furthermore, the tested materials differed not only in filler characteristics but also in resin matrix composition, making it difficult to distinguish the individual contribution of each compositional variable to the observed properties. Therefore, the findings should be interpreted as reflecting the overall formulations of the commercial resin composites rather than the effect of filler content alone. Finally, although the hydrothermal accelerated aging protocol provides a rapid method for evaluating material stability, its relatively short duration may not fully reproduce the complex long-term aging conditions encountered in the oral environment.

## 5. Conclusions

Within the limitations of this in vitro study, the choice of resin composite should consider the balance between mechanical performance, polymerization behavior, and surface properties. CLEARFIL AP-X demonstrated the highest mechanical strength but exhibited lower surface gloss and greater surface roughness. CLEARFIL MAJESTY ES-Flow showed superior wear resistance and surface gloss but generated higher shrinkage stress. CLEARFIL MAJESTY ES-2 provided intermediate mechanical and surface properties but exhibited the lowest degree of monomer conversion among the tested materials. These findings are based on laboratory testing, and further clinical studies are needed to determine their clinical relevance.

## Figures and Tables

**Table 1:** Materials used in the study.

Materials (Lot No.)	Abbreviation	Composition	Manufacturer
CLEARFIL AP-X (770768)	AP-X	Bis-GMA, TEGDMA, silanated barium glass filler, silanated silica filler, silanated colloidal silica, DL-Camphorquinone (filler size 0.1–15 μm, average; 3 μm) 71 vol% (85 wt.%).	(Kuraray Medical Co., Tokyo, Japan)
CLEARFIL MAJESTY ES-Flow (7K0380)	ES-Flow	TEGDMA, hydrophobic aromatic dimethacrylate, silanated barium glass filler, silanated silica (filler size 0.18–3.5 μm) 59 vol% (75 wt.%).	
CLEARFIL MAJESTY ES-2 (750225)	ES-2	Bis-GMA, hydrophobic aliphatic dimethacrylate, hydrophobic aromatic dimethacrylate, silanated barium glass filler, pre-polymerized organic filler including nano filler (nano fillers size 1–100 nm) 66 vol% (78 wt.%).	

**Table 2:** Elemental structure of the investigated resin composite determined using EDS analysis.

Material	Elemental Composition by Weight %
AP-X	C 4.86%, O 45.01%, Al 4.13%, Si 22.52%, Cl 0.06%, Ba 23.42%
ES-Flow	C 7.60%, O 50.60%, Na 0.59%, Al 3.89%, Si 21.84%, Ba 15.47%
ES-2	C 10.02%, N 1.17%, O 55.77%, Al 1.92%, Si 19.95%, Cl 0.06%, Ba 11.10%

## Data Availability

The original contributions presented in this study are included in the article.
